# Double-edged-sword effect of bisphosphonates on the osteogenic differentiation of human periodontal ligament stem cells

**DOI:** 10.3389/fphar.2026.1752252

**Published:** 2026-03-03

**Authors:** Mengyu Li, Jiajia Wang, Hanjin Ruan, Zhouyang Wang, Shaoyi Wang, Yue He, Zhiyuan Zhang

**Affiliations:** 1 Department of Oral-maxillofacial Head and Neck Oncology, Shanghai Ninth People’s Hospital, Shanghai Jiao Tong University School of Medicine, Shanghai, China; 2 College of Stomatology, Shanghai Jiao Tong University, Shanghai, China; 3 National Center for Stomatology, National Clinical Research Center for Oral Diseases, Shanghai, China; 4 Shanghai Key Laboratory of Stomatology, Shanghai, China; 5 Shanghai Research Institute of Stomatology, Shanghai, China; 6 Research Unit of Oral and Maxillofacial Regenerative Medicine, Chinese Academy of Medical Sciences, Shanghai, China; 7 Department of Prosthodontics, Shanghai Ninth People’s Hospital, Shanghai Jiao Tong University School of Medicine, Shanghai, China; 8 Department of Oral Surgery, Shanghai Ninth People’s Hospital, Shanghai Jiao Tong University School of Medicine, Shanghai, China

**Keywords:** bisphosphonates, bone regeneration, MAPK, osteogenesis, periodontal ligament stem cells, Wnt/β-catenin

## Abstract

Bisphosphonates (BPs), widely used anti-resorptive agents for osteoporosis and cancer-related bone metastasis, can paradoxically contribute to medication-related osteonecrosis of the jaw (MRONJ). Our previous work showed that periodontal ligament stem cells (PDLSCs) from MRONJ patients display severely impaired osteogenesis; however, how BPs directly regulate PDLSC function remains unclear. In this study, human PDLSCs were exposed to graded concentrations of zoledronate (ZOL, 0.01–10 μM) to characterize dose-dependent effects on cell viability, apoptosis, and osteogenic differentiation. High-dose ZOL markedly reduced proliferation, induced apoptosis, and strongly inhibited osteogenesis. In contrast, low-dose ZOL promoted osteogenic differentiation *in vitro*, enhanced mineralization, and increased ectopic bone formation *in vivo*. Transcriptomic and molecular analyses revealed that ZOL activated Wnt/β-catenin and MAPK signaling, and blockade of either pathway attenuated the osteogenic enhancement. These findings demonstrate a double-edged-sword effect of BPs on PDLSCs: low-dose ZOL enhances osteogenesis through coordinated activation of Wnt/β-catenin and MAPK pathways, whereas high-dose exposure is cytotoxic and suppresses regenerative potential. The results underscore the necessity of precise BP dose control to maximize periodontal regeneration while minimizing MRONJ risk.

## Introduction

Periodontal ligament stem cells (PDLSCs) represent a pivotal resource for regenerative therapies due to their multipotency and immunomodulatory properties (Tomokiyo et al., 2019). However, their osteogenic potential is frequently compromised in pathological microenvironments, particularly in the periodontal niche of patients with medication-related osteonecrosis of the jaw (MRONJ)—a devastating complication linked to long-term bisphosphonates (BPs) therapy for osteoporosis and metastatic bone disease (Ruggiero et al., 2014; Kishimoto et al., 2019; Endo et al., 2017).

Our previous study revealed that PDLSCs isolated from MRONJ patients (MRONJ-PDLSCs) display markedly reduced proliferative capacity, impaired adhesion and migratory abilities, and a significantly diminished osteogenic differentiation potential (Limones et al., 2020). While these findings implicate the local microenvironment as a key factor in impairing PDLSC-mediated periodontal regeneration in MRONJ, the underlying molecular mechanisms have yet to be fully elucidated.

Accumulating clinical evidence indicates a pronounced dose-dependent relationship between BPs exposure and the development of MRONJ (Ruksakiet et al., 2025; Moreno Rabie et al., 2023). Oncology patients, who approximately receive a ten-fold higher dosage of BPs than those with osteoporosis, exhibit a significantly higher incidence of MRONJ (Jiang et al., 2024). Although the exact pathogenic mechanisms remain unclear, cumulative BP dosage is widely regarded as a key determinant. Given that BPs, especially Zoledronate (ZOL) —a third-generation nitrogen-containing bisphosphonate compound with potent anti-resorptive properties—are preferentially deposited in alveolar bone and adjacent periodontal tissues, suggesting that BPs may exert a direct influence on the biological behavior of PDLSCs (Akoury et al., 2019; Alsalleeh et al., 2014). However, existing studies have predominantly focused on the systemic effects of BPs or the pathological features of MRONJ lesion, leaving the dose-dependent influence of BPs on healthy PDLSCs largely unexplored (Yarom et al., 2019). Specifically, it remains unclear whether low-dose BPs may preserve or even enhance the osteogenic capacity of PDLSCs, while high-dose exposure induces cytotoxicity and osteonecrosis, as observed clinically.

To simulate clinically relevant exposure, ZOL concentrations of 0.01–10 μM were selected based on reported serum and bone-surface levels after systemic administration (Raccor et al., 2013; Chen et al., 2002; Vassaki et al., 2022), reflecting physiologically and pathologically relevant conditions corresponding to osteoporotic versus oncologic dosing. This study systematically investigated the dose-dependent effects of ZOL on PDLSC osteogenic differentiation *in vitro* and *in vivo*. By modeling exposures from physiological to oncologic levels, we aimed to determine whether low-dose ZOL enhances osteogenesis while high-dose induces cytotoxicity. We further explored underlying mechanisms, focusing on Wnt/β-catenin–MAPK crosstalk governing PDLSC osteogenic commitment, to elucidate the molecular basis of bisphosphonate-modulated regeneration and MRONJ pathogenesis.

## Materials and methods

### Cell culture

Human periodontal ligament stem cells (PDLSCs) were isolated from freshly extracted orthodontic premolars obtained from three healthy donors (two females and one male, aged 14–18 years) with written informed consent. The experimental protocol was approved by the Ethics Committee of the Ninth People’s Hospital (SH9H-2020-T36-2). PDLSCs were cultured and identified following previously reported protocols (Moshaverinia et al., 2014). Cells from different donors were not pooled; experiments were conducted independently for each donor to account for biological variability. Passages three to five were used for all experiments.

### Zoledronate treatment

Zoledronate (ZOL) was obtained from Novartis Pharmaceuticals. PDLSCs were treated with ZOL at 0.01, 0.1, 0.5, 1, and 10 µM. The ZA concentrations used in this study were chosen based on human pharmacokinetic studies, ensuring that low-dose conditions (0.01–0.5 µM) approximate systemic and early bone surface exposures in osteoporosis, while higher-dose conditions (1–10 µM) model local skeletal accumulation observed in oncology patients receiving repeated high-dose therapy. In humans, a standard 4 mg IV infusion for osteoporosis yields peak plasma concentrations of 0.11–0.26 µM. Rapid skeletal uptake reduces plasma levels within 24 h, while local bone surface concentrations are modestly higher, supporting the use of 0.01–0.5 µM to mimic systemic and early bone exposures in osteoporotic patients (Skerjanec et al., 2003; Raccor et al., 2013; Cai et al., 2018). Oncology patients with bone metastases receive more frequent, higher-dose regimens (4 mg every 4 weeks), resulting in repeated high local skeletal concentrations (0.4–4.6 µM) due to both increased dosing and higher bone turnover (Ruggiero et al., 2014). Accordingly, 1–10 µM ZOL was employed to model high local exposure conditions associated with clinical MRONJ risk. Thus, the 0.01–10 µM range covers physiologically relevant plasma levels and potential local enrichment on bone surfaces.

### Cell proliferation

PDLSCs (5 × 10^3^ cells/mL) were seeded into 96-well plate and treated with ZOL (0.01, 0.1, 0.5, 1, 10 μM) or vehicle (0.9% NaCl). Cell proliferation was assessed at 1, 3, 5 and 7 days using CCK-8 assay.

### Cell apoptosis

PDLSCs were seeded into 6-well plate and treated with ZOL for 72 h. Cell apoptosis was analyzed using FITC Annexin V Apoptosis Detection Kit and Cell Death Fluorescein Detection Kit.

### Morphological analysis

PDLSCs were seeded in 12-well plate and treated with ZOL for 72 h. Morphology was observed by phase-contrast microscopy, and cytoskeleton stained with TRITC-phalloidin (YEASEN) was imaged under fluorescence microscopy.

### Alkaline phosphatase (ALP) staining

PDLSCs (5 × 10^3^ cells/well) were seeded in 24-well plates and allowed to attach overnight. Cells were then continuously treated with ZOL at different concentrations for 72 h. After osteogenic induction for 7 days, ALP staining was performed with BICP/NBT Kit. Then cells were lysed with 1% Triton X-100 and subjected to three cycles of freezing and thawing. ALP activity was measured at 405 nm and normalized to protein content. ZOL was continuously present in the culture medium (total 10 days).

### Alizarin red staining (ARS)

Cell treatment and processing steps were carried out following the same protocol as the ALP staining. PDLSCs were induced in the osteogenic media for 14 days, then cells were fixed in 4% paraformaldehyde and stained with 1% Alizarin Red. Alizarin Red dye was extracted with 400 µL of 10% cetylpyridinium chloride for 15 min and quantified at 562 nm.

### Quantitative real-time PCR (qRT-PCR)

PDLSCs were seed in 6-well plate and treated with ZOL for 72 h. Then total RNA was isolated using with Trizol, reverse transcribed by Superscript II. Real-time PCR was performed with Light Cycler^®^ 480 II. β-Actin was used to normalize gene expression. Primers used in this study are shown in Supplementary Table S1.

### Immunofluorescence

PDLSCs were treated with ZOL for 72 h, fixed, permeabilized, and blocked, then incubated with Runx2 and OCN antibodies (overnight, 4 °C), followed by secondary antibodies and DAPI staining. Fluorescence images were captured using fluorescence microscope.

### Ectopic bone regeneration *in vivo*

Constructs were prepared by seeding 5 × 10^7^ PDLSCs onto β-tricalcium phosphate (β-TCP) cuboids and transplanted into aseptically created subcutaneous pockets in 6-week-old male nude mice. A total of 30 constructs were randomly assigned to divided into six groups: control (β-TCP + PDLSCs without ZOL), and ZOL-treated groups at 0.01, 0.1, 0.5, 1, and 10 µM (n = 5 per group, one construct per mouse). After 12 weeks, the implants were harvested and fixed in 4% paraformaldehyde for histological analysis. All animal experimental procedures were approved by the Experimental Animal Welfare and Ethics Branch of Shanghai Ninth People’s Hospital (SH9H-2020-A670-1).

### Histological analysis

Transplants were decalcified in 10% EDTA for 2 months, then washed, dehydrated, and embedded in paraffin. The sections were cut at 5 μm thickness and stained with H&E and Masson staining. The sections were also evaluated by immunofluorescence, as described previously (Limones et al., 2020).

### cDNA microarray analysis

Periodontal ligament stem cells (PDLSCs) from a single donor were treated with ZOL (0.5 µM) or vehicle control for 3 days (n = 3, biological replicates per group). Gene expression profiling was performed using Affymetrix GeneChips. Raw data were normalized by the RMA method in R, and differentially expressed genes were identified as those showing a ≥2-fold change with p < 0.05. Gene Ontology (GO) and Kyoto Encyclopedia of Genes and Genomes (KEGG) enrichment analyses were conducted using the clusterProfiler package (v3.16.0). P-values were adjusted using the Benjamini–Hochberg (BH) method, and pathways with an adjusted p ≤ 0.05 were considered significant.

### Western blotting

Cells were lysed in RIPA buffer, and proteins were extracted, separated by 10% SDS-PAGE, and transferred to membranes. After blocking, membranes were incubated with primary antibodies (overnight, 4 °C) and secondary antibodies (1 h, RT). GAPDH served as a loading control. Signals were visualized with ChemiDoc XRS and quantified using ImageJ.

### Statistical analysis

All the data were obtained from at least three independent experiments. The values were presented as mean ± standard deviation (SD). The Student’s t-test and one-way ANOVA were used to perform statistical analyses using GraphPad Prism 7.0 (GraphPad Software, United States). *P* < 0.05 was considered statistically significant.

## Results

### Characterization and multilineage differentiation potential of PDLSCs

**FIGURE 1:**
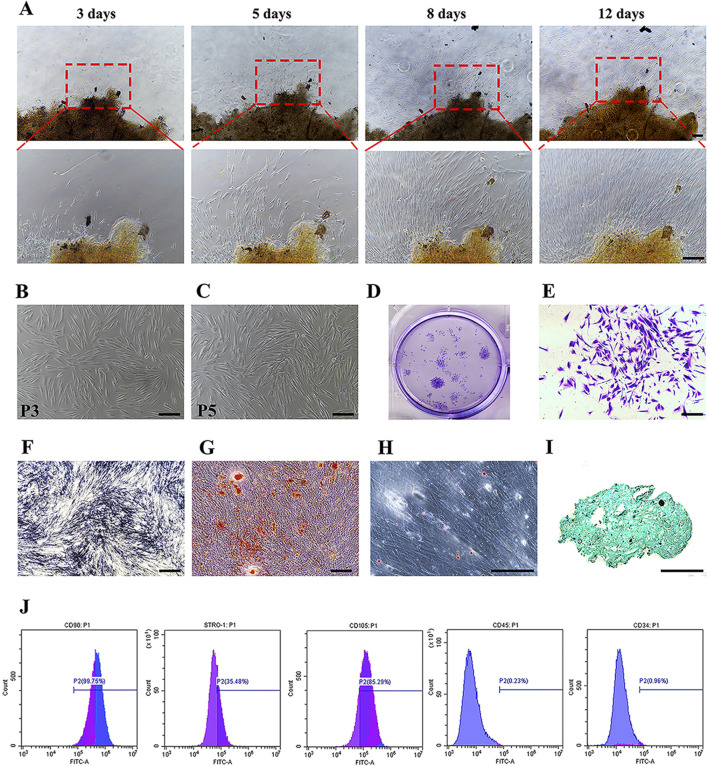
Characterization and multilineage differentiation potential of PDLSCs. **(A)** Representative images showing PDLSCs migrating from PDL tissues and expanding between days 3 to 12 after primary seeding. **(B,C)** Morphology of PDLSCs at passage 3 **(B)** and passage 5 **(C)**, displaying typical spindle-shaped, fibroblast-like appearance. **(D,E)** Colony-forming unit-fibroblast (CFU-F) assays revealed clonogenic potential of PDLSCs, as shown by crystal violet-stained colonies. **(F–I)** Multilineage differentiation capacity of PDLSCs: osteogenic differentiation assessed by ALP staining **(F)** and Alizarin Red staining of calcified nodules **(G)**; adipogenic differentiation by Oil Red O staining of lipid droplets **(H)**; and chondrogenic differentiation by Alcian Blue staining **(I,J)** Flow cytometry data of mesenchymal surface markers on PDLSCs (Scale bars: 100 μm).

Primary adherent cells migrated from the periodontal ligament (PDL) tissues within 3–5 days, proliferating progressively to 90% confluence within 3–12 days and exhibiting spindle-shaped, fibroblast-like morphology (Figure 1A). Cells at passages 3–5 maintained this morphology (Figures 1B,C) and were used for experiments. PDLSCs exhibited colony-forming ability (Figures 1D,E) and multipotency: osteogenic differentiation was confirmed by ALP staining and mineralized nodule formation (Figures 1F,G), adipogenic differentiation by lipid droplet accumulation (Figure 1H), and chondrogenic differentiation by Alcian blue staining (Figure 1I). Flow cytometry showed strong expression of MSC markers STRO-1, CD90, and CD105, with minimal hematopoietic marker CD34 and CD45 expression (Figure 1J).

### Dual effect of ZOL on the proliferation, apoptosis and morphology of PDLSCs

**FIGURE 2:**
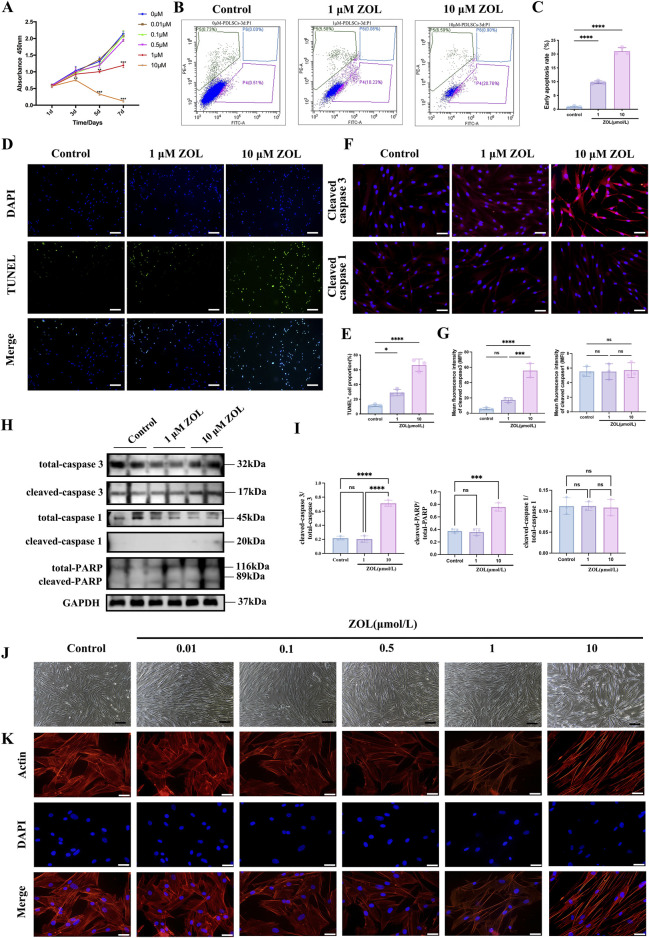
Effects of different dosages of ZOL on the proliferation, apoptosis and morphology of PDLSCs. **(A)** The growth curves of PDLSCs in culture with ZOL (0.01, 0.1, 0.5, 1 and 10 μM). At 1, 3, 7 days. **(B)** The effect of ZOL on PDLSCs apoptosis were determined by flow cytometric analysis. **(C)** Quantitative analysis of the rate of early apoptotic cells in different groups. **(D)** Photos depicting DAPI, TUNEL, and merge staining in each group were also used to assessed cell apoptosis (Scale bar: 100 μm). **(E)** Quantitative analysis of the TUNEL^+^ cells in different groups. **(F)** Immunofluorescence staining of cleaved caspase-3 and caspase-1 (Scale bar: 50 μm), and **(G)** quantitative analysis. **(H)** Representative western blots of cleaved caspase-3, PARP, and caspase-1 in PDLSCs treated with 1µM and 10 µM ZOL. GAPDH served as a loading control. **(I)** Quantitative analysis of western blots data. **(J)** The changes of morphology in PDLSCs treated with ZOL under inverted phase-contrast optical microscope (Scale bar: 200 μm). **(K)** Immunofluorescence assay for actin fiber evaluation under fluorescence microscope (Scale bar: 50 μm). Data represent the mean values (n = 3). Error bars, SDs from the mean values (*P < 0.05, **P < 0.01, ***P < 0.001).

High concentrations ZOL (1 and 10 μM) significantly suppressed proliferation at 72 h, with 10 μM showing the strongest effect, while ≤0.5 μM had minimal impact (Figure 2A). To further determine whether the observed the inhibition of proliferation was associated with ZOL-induced cell deaths, apoptotic activity was assessed using complementary approaches. Flow cytometry analysis using Annexin V-FITC/PI staining, revealed a significant increase in the proportion of early apoptotic cells (Annexin V^+^/PI^−^) in both 1 μM and 10 μM ZOL-treated groups compared with controls, while the late apoptotic/necrotic population (Annexin V^+^/PI^+^) showed no statistically significant change (Figures 2B,C). Consistently, TUNEL staining demonstrated a markedly higher percentage of TUNEL^+^ cells in the high-dose ZOL groups (Figures 2D,E), indicating increased DNA fragmentation.

To further distinguish apoptosis from pyroptosis, we examined key molecular markers of distinct cell death pathways. Immunofluorescence staining and Western blot analysis (Figures 2F–I) showed robust upregulation of cleaved caspase-3 and cleaved PARP in PDLSCs treated with high-dose ZOL, confirming activation of the canonical apoptotic cascade. In contrast, expression levels of caspase-1, a central mediator of pyroptosis, remained unchanged across treatment groups, indicating that inflammatory/pyroptotic cell death was not activated under these conditions. These molecular findings are concordant with the flow cytometry results, which showed selective induction of early apoptosis without a concomitant increase in late apoptotic/necrotic cells. Morphological assessment further supported these observations. Control and ≤0.5 μM ZOL-treated PDLSCs retained typical spindle-shaped fibroblastic morphology with well-organized F-actin cytoskeletons (Figures 2J,K). In contrast, cells exposed to 1 μM and 10 μM ZOL exhibited pronounced morphological alterations, including cell shrinkage, irregular contours, and disrupted actin filament organization, which are characteristic of apoptotic cytoskeletal remodeling.

Collectively, these results demonstrate that ZOL exerts a dose-dependent cytotoxic effect on PDLSCs. At concentrations ≥1 μM, ZOL suppresses proliferation and induces classic apoptosis—characterized by early apoptotic signaling, caspase-3/PARP activation, and cytoskeletal disruption—without engaging pyroptotic pathways. These findings establish apoptosis as the primary mode of ZOL-induced cell death in PDLSCs and provide a mechanistic basis for dose selection in subsequent experiments.

### Dual effect of ZOL on the osteogenesis of PDLSCs *in vitro*

**FIGURE 3:**
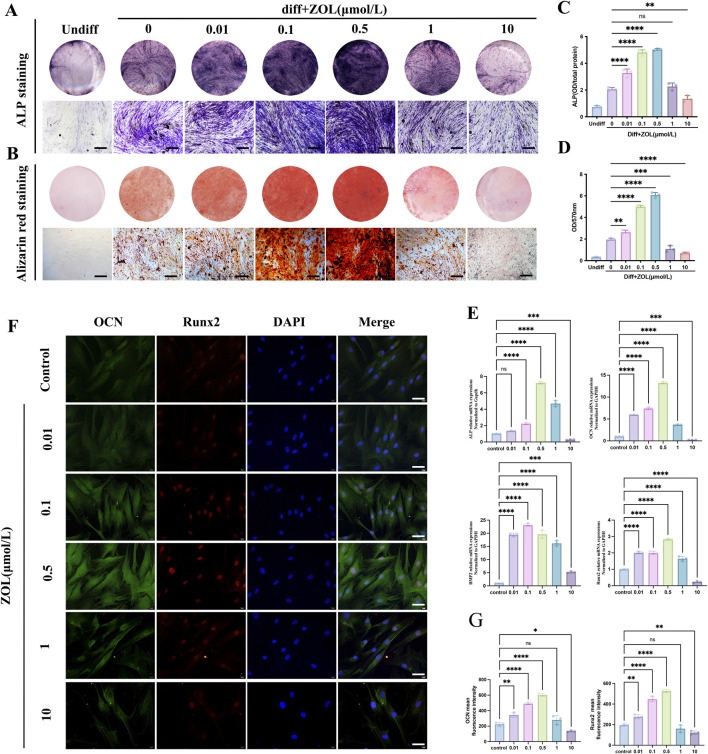
The effects of ZOL on the osteogenic differentiation of PDLSCs *in vitro*. **(A)** Entire plate views and micrographs of ALP staining at 7 days and **(B)** Alizarin Red staining at 14 days (Scale bar: 100 μm). **(C)** Quantitative evaluation of ALP activity. **(D)** Quantification of the Alizarin Red staining results. **(E)** The mRNA expression of ALP, BMP2, OCN, and Runx2 in PDLSCs of all groups was subjected to real-time PCR analysis. **(F)** Immunofluorescence staining for OCN and RUNX2 on PDLSCs in each groups after 7 days’ osteogenic differentiation (Scale bar: 50 μm), and **(G)** quantitative analysis. Data represent the mean values (n = 3). Error bars, SDs from the mean values (*P < 0.05, **P < 0.01, ***P < 0.001).

ALP staining on day 7 (Figure 3A) and alizarin red staining on day 14 (Figure 3B) revealed a biphasic response: low concentrations of ZOL (≤0.5 μM) enhanced osteogenic differentiation in a dose-dependent manner, whereas high concentrations (10 μM) markedly suppressed osteogenic activity. This dose-dependent dual effect was further supported by quantitative analysis of ALP activity (Figure 3C) and alizarin red content (Figure 3D), both of which peaked at 0.5 μM and declined sharply after 1 μM.

To further elucidate the molecular mechanisms underlying the biphasic effect, we assessed transcriptional levels of key osteogenic genes by RT-qPCR. ALP, RUNX2, and OCN peaked at 0.5 μM ZOL, whereas BMP2 peaked at 0.1 μM. High-dose ZOL (10 μM) significantly suppressed all four genes. In the 1 μM group, mRNA levels were lower than 0.5 μM but remained higher than control (Figure 3E). Consistently, immunofluorescence showed highest OCN and Runx2 at 0.5 μM, moderate reduction at 1 μM, and marked suppression at 10 μM (Figure 3F). Collectively, ZOL regulates PDLSC osteogenesis in a dose-dependent, biphasic manner: low concentrations (≤0.5 μM) enhance differentiation, high concentrations (≥10 μM) inhibit it, and 1 μM represents a transitional threshold. Thus, 0.5 μM was selected as the optimal concentration for subsequent assays.

### Comparative ectopic osteogenesis *in vivo*

**FIGURE 4:**
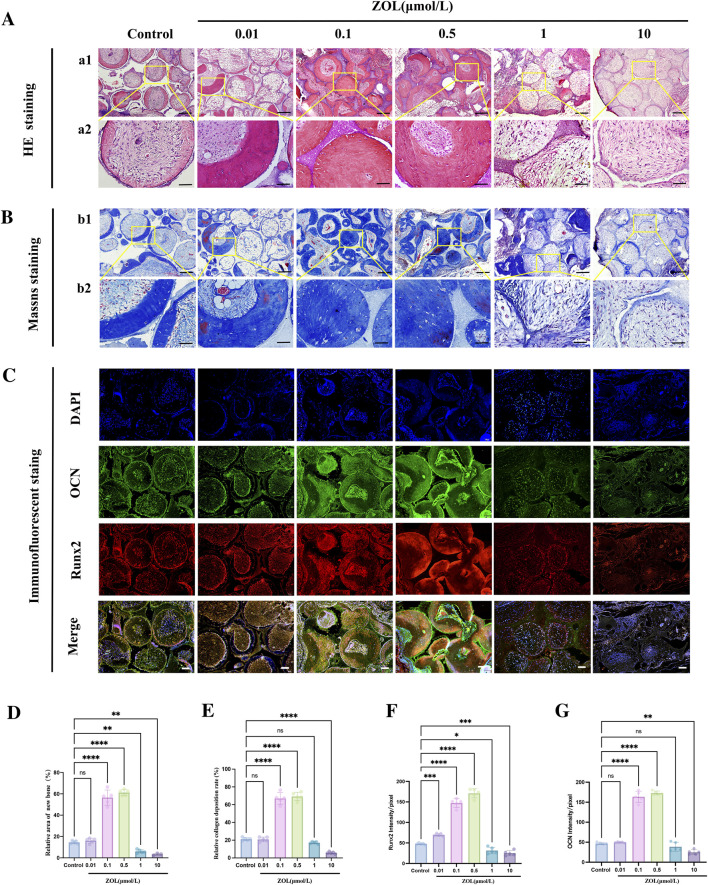
The effects of ZOL on ectopic bone regeneration capacity of PDLSCs *in vivo*. **(A)** Histologic analysis of transplants was performed by H&E staining showing new bone formation at different groups (a1, Scale bar: 100 μm; a2, Scale bar: 30 μm). **(B)** Representative images of Masson staining of the transplants in each group showing collagen deposition (b1, Scale bar: 100 μm; b2, Scale bar: 30 μm). **(C)** Immunofluorescence images displayed the expression of OCN and Runx2 in each group (Scale bar: 100 μm). **(D)** Quantification results of new bone formation rate and **(E)** the percentage of collagen in each group. **(F,G)** Quantitative evaluation of the expressions of OCN and Runx2 using Image(J). Data represent the mean values (n = 5). Error bars, SDs from the mean values (*P < 0.05, **P < 0.01, ***P < 0.001).

Further evaluate the *in vivo* effects of ZOL on the osteogenesis of PDLSCs, β-TCP scaffolds seeded with PDLSCs were implanted subcutaneously into nude mice. After 12 weeks, these implants were harvested for histological analysis. H&E staining (Figure 4A) demonstrated a marked increase in new bone formation in the 0.1 μM and 0.5 μM ZOL groups compared with the control group. In contrast, new bone formation was profoundly diminished in the 1 μM and 10 μM groups, showing a statistically significant reduction compared with the control group (Figure 4D). Masson’s trichrome staining (Figure 4B) revealed abundant, well-organized collagen fibers in the 0.1 μM and 0.5 μM ZOL groups. In contrast, 1 μM and 10 μM groups displayed markedly reduced and sparse collagen deposition. Quantitative analysis of collagen fraction confirmed a significant increase in collagen content in the 0.1 μM and 0.5 μM groups compared to the control (Figure 4E). Immunofluorescence staining for OCN and Runx2 (Figure 4C) further validated these observations. Both the intensity and area of positive staining were markedly elevated in the 0.1 μM and 0.5 μM groups, moderate in the control group, and substantially diminished in the 1μM and 10 μM groups (Figures 4F,G).

These results demonstrate that ZOL at 0.1 μM and 0.5 μM significantly promotes ectopic bone formation of PDLSCs *in vivo*, whereas 0.01 μM shows negligible effect, and higher concentrations (1 μM and 10 μM) markedly suppress their osteogenesis.

### Activation of Wnt/β-catenin and MAPK signaling pathway in ZOL-treated PDLSCs

**FIGURE 5:**
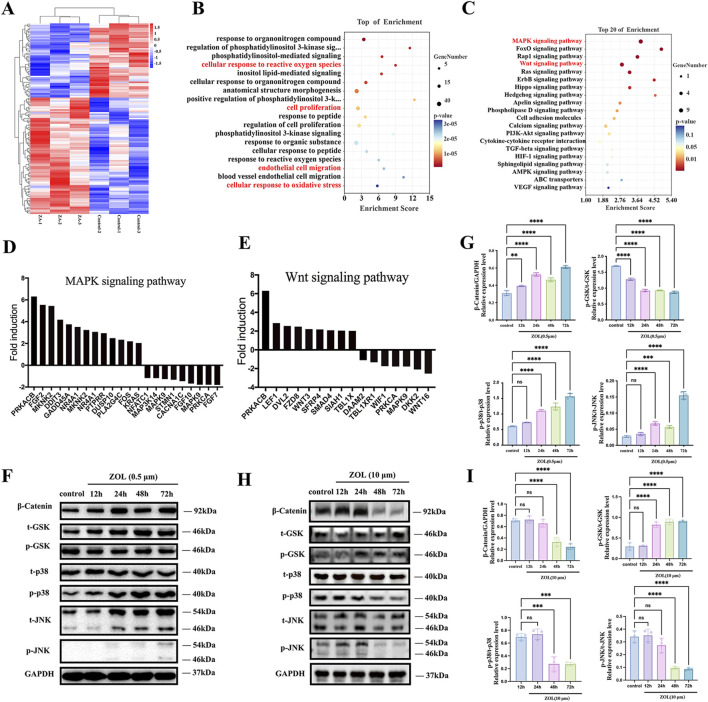
Dose-dependent regulation of Wnt/β-catenin and MAPK signaling pathways in ZOL-treated PDLSCs. **(A)** Heatmap of differentially expressed genes (fold change ≥2) in PDLSCs treated with 0.5 μM ZOL versus control. **(B,C)** GO and KEGG enrichment analyses showing the top 20 enriched biological processes and signaling pathways, with Wnt and MAPK pathways highlighted. **(D,E)** Differentially expressed genes involved in Wnt/β-catenin and MAPK signaling (fold change ≥2). **(F)** Representative western blots of β-catenin, phosphorylated GSK-3β (p-GSK), phosphorylated p38 (p-p38), and phosphorylated JNK (p-JNK) in PDLSCs treated with low-dose ZOL (0.5 µM) ZOL for the indicated times. GAPDH served as a loading control. **(G)** Quantitative densitometric analysis of protein expression and phosphorylation levels in low-dose ZOL, normalized to corresponding total protein levels or GAPDH. **(H)** Representative Western blot images of PDLSCs treated with high-dose ZOL (10 µM), showing absence of sustained β-catenin stabilization, increased p-GSK levels, and suppression of p38 and JNK phosphorylation at later time points (48–72 h). **(I)** Quantitative densitometric analysis corresponding to high-dose ZOL, confirming inhibition of Wnt/β-catenin and MAPK pathway activation. Data represent the mean values (n = 3). Error bars, SDs from the mean values (*P < 0.05, **P < 0.01, ***P < 0.001).

To explore mechanisms of ZOL-induced osteogenesis in PDLSCs, we performed an Affymetrix Gene Expression Array. Differentially expressed genes exhibiting more than 2-fold up or downregulation were identified in the heat map (Figure 5A). GO and KEGG analyses identified the top 20 enriched biological processes and signaling pathways (Figures 5B,C). Among these, the MAPK and Wnt signaling pathways as prominent signaling cascades potentially mediating ZOL-induced osteogenic differentiation. Differentially expressed genes involved in these two pathways, showing greater than twofold changes, are listed in Figures 5D,E.

To verify the involvement of the Wnt/β-catenin and MAPK pathways in ZOL-promoted osteogenesis in PDLSCs, we performed Western blot analysis under both low-dose and high-dose ZOL conditions (Figures 5F–I). Under osteogenic-promoting conditions (0.5 µM ZOL), β-catenin protein expression was significantly increased as early as 12 h and remained consistently elevated from 12 to 72 h (Figure 5F). Concomitantly, phosphorylated GSK (p-GSK) levels were markedly decreased, suggesting inhibition of GSK kinase activity and consequent stabilization of β-catenin. Activation of MAPK signaling was evident after 24 h of ZOL exposure, as indicated by sustained and pronounced increases in phosphorylation of p38 and JNK from 24 to 72 h, with peak activation observed at 72 h. Quantitative densitometric analysis (Figure 5G) confirmed significant elevations in β-catenin, p-p38, and p-JNK levels, together with a robust reduction in p-GSK expression. In contrast, high-dose ZOL treatment (10 µM) failed to elicit sustained activation of either pathway (Figures 5H,I). At early time points (12–24 h), β-catenin abundance and phosphorylation levels of p38 and JNK remained largely unchanged compared with controls. However, prolonged exposure (48–72 h) resulted in a significant reduction of β-catenin protein, accompanied by increased p-GSK levels, indicative of enhanced β-catenin degradation and suppression of canonical Wnt signaling. In parallel, phosphorylation of p38 and JNK was markedly decreased at 48 and 72 h, while total protein levels remained unchanged, reflecting inhibition of MAPK pathway activation rather than altered protein expression. Quantitative analysis further substantiated these inhibitory effects. Together, these data demonstrate a dose-dependent divergence in signaling responses to ZOL: low-dose ZOL induces coordinated and sustained activation of Wnt/β-catenin and MAPK pathways, whereas high-dose ZOL suppresses both signaling axes. This comparative analysis establishes high-dose ZOL as an effective negative control for pathway activation and supports the conclusion that selective activation of Wnt/β-catenin–MAPK signaling underlies the osteogenic-promoting effects of low-dose ZOL in PDLSCs.

### Inhibition of the Wnt/β-catenin and MAPK signaling pathways attenuated ZOL-induced osteogenic promotion in PDLSCs

**FIGURE 6:**
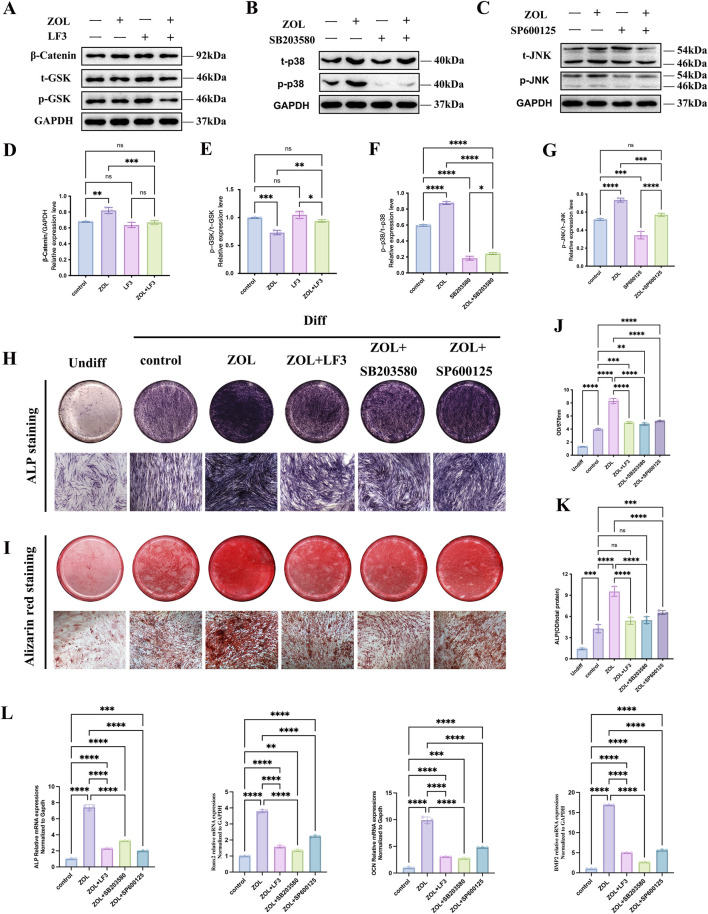
Inhibition of Wnt/β-catenin and MAPK signaling attenuates ZOL-induced osteogenesis in PDLSCs. **(A–C)** Representative western blots showing β-catenin, phosphorylated p38 (p-p38), and phosphorylated JNK (p-JNK) levels in PDLSCs pretreated with LF3 (β-catenin inhibitor), SB203580 (p38 inhibitor), or SP600125 (JNK inhibitor) for 24 h, followed by 0.5 μM ZOL stimulation for 24 h. GAPDH served as a loading control. **(D–G)** Quantitative densitometric analysis confirming significant suppression of β-catenin, p-p38, and p-JNK activation by their respective inhibitors. **(H)** ALP activity assay and **(I)** Alizarin Red S staining showing reduced early and late osteogenic differentiation upon pathway inhibition in ZOL-treated PDLSCs. **(L)** qRT-PCR analysis of osteogenic marker genes (BMP2, RUNX2, ALP, and OPN) demonstrating downregulation after inhibition of β-catenin, p38, or JNK signaling. Error bars, SDs from the mean values (*P < 0.05, **P < 0.01, ***P < 0.001).

To determine whether the Wnt/β-catenin and MAPK signaling pathways mediate the osteogenic effects of ZOL on PDLSCs, we selectively inhibited β-catenin (LF3), p38 (SB203580), and JNK (SP600125). PDLSCs were pretreated with each inhibitor before ZOL stimulation. Western blot analysis revealed that LF3, SB203580, and SP600125 effectively reduced β-catenin accumulation, as well as the phosphorylation of p38, and JNK, respectively, after 24 h (Figures 6A–C). Quantitative densitometry confirmed significant suppression of pathway activation (Figures 6D–G). Functionally, inhibition of either the Wnt/β-catenin or MAPK pathways markedly reduced ALP activity (Figure 6H) and mineralized nodule formation (Figure 6I) in ZOL-treated PDLSCs. Consistently, real-time PCR analysis showed that blockade of these pathways significantly downregulated the expression of osteogenic marker genes (BMP2, RUNX2, ALP, OCN) (Figure 6L). Collectively, these findings demonstrate that activation of the Wnt/β-catenin and MAPK pathways plays a positive and functional role in mediating the ZOL-induced osteogenic differentiation of PDLSCs.

## Discussion

Medication-related osteonecrosis of the jaw (MRONJ) has emerged as a significant clinical concern in patients receiving nitrogen-containing bisphosphonates (BPs), such as zoledronate (ZOL), for osteoporosis, metastatic bone disease, or other skeletal disorders (Yarom et al., 2019). Although MRONJ prevalence is relatively low in the general osteoporotic population (Wong et al., 2025), it rises substantially in oncology patients receiving high-dose or long-term intravenous BPs (Bertoldo et al., 2025). The condition features exposed necrotic bone, delayed healing, and susceptibility to infection, often triggered by dental extractions or other oral surgical procedures. Its multifactorial pathogenesis—including suppression of bone turnover, antiangiogenic effects, immune modulation, and microbial colonization—raises concerns regarding BP use in dental and maxillofacial practice (Lee et al., 2025; Roato et al., 2023; Srivichit et al., 2022).

Paradoxically, BPs have also been reported to possess pro-osteogenic properties, by stimulating osteoblast differentiation, inhibiting osteoclast-mediated resorption, and modulating signaling cascades related to bone metabolism (Zhou et al., 2011; Teixeira et al., 2019; Cremers et al., 2020). This creates a translational dilemma: while systemic administration at therapeutic doses for skeletal disease can predispose to MRONJ, localized and low-dose delivery might serve as a potent adjuvant for bone regeneration in periodontal and implant therapies. The dual role of BPs thus represents a “double-edged sword,” where the therapeutic window is narrow and the balance between efficacy and toxicity is delicate.

In the present study, we sought to clarify the molecular mechanisms by which ZOL influences osteogenesis in PDLSCs and to define a dose range that maximizes regenerative potential while minimizing cytotoxicity. Our results demonstrated a double-edged-sword effect: low concentrations ZOL (0.5 μM) significantly enhanced osteogenic differentiation *in vitro* and promoted ectopic bone formation *in vivo*, whereas higher concentrations (≥10 μM) suppressed proliferation, induced apoptosis, and reduced mineralization capacity.

The cytotoxic effects observed at higher ZA concentrations are not attributable to nonspecific chemical toxicity, but rather reflect a well-established, bisphosphonate-specific and target-mediated mechanism. Nitrogen-containing bisphosphonates, including zoledronate, exert their biological effects through selective inhibition of farnesyl pyrophosphate synthase (FPPS) in the mevalonate pathway, leading to depletion of essential isoprenoid intermediates and impaired prenylation of small GTPases such as Ras, Rho, and Rac. This disruption compromises cytoskeletal organization, mitochondrial integrity, and survival signaling, ultimately triggering apoptosis (Dunford et al., 2006; Gadelha et al., 2020).

Although initially designed to target osteoclasts, accumulating evidence demonstrates that excessive or prolonged exposure to nitrogen-containing bisphosphonates induces dose-dependent cytotoxicity in a broad range of non-osteoclastic cells, including mesenchymal stem cells, periodontal ligament stem cells, osteoblasts, and endothelial cells (Açil et al., 2012; Budzinska et al., 2023; Guirguis et al., 2023). These cellular responses—characterized by reduced proliferation, cytoskeletal disruption, caspase activation, and apoptosis—are consistently observed at micromolar concentrations and closely mirror the phenotypes identified in our high-dose ZA (10 μM) group. Importantly, such BP-specific cytotoxicity has been directly implicated in the pathogenesis of MRONJ, where excessive local drug accumulation impairs resident stem cell survival, angiogenesis, and tissue regeneration (Ziebart et al., 2011; Walter et al., 2011). Thus, the high-dose cytotoxic effects observed in our study represent a pharmacologically relevant and disease-associated phenomenon, reinforcing the concept of a narrow therapeutic window underlying the “double-edged sword” effect of bisphosphonates.

These findings mirror clinical observations that localized, short-term BP exposure may have anabolic effects on bone, whereas chronic systemic exposure at higher doses increases MRONJ risk (Vuorimies et al., 2017). These observed dose-dependent effects are further supported by clinically relevant pharmacokinetics of ZA. After standard intravenous infusion (4 mg), peak plasma concentrations in osteoporosis patients typically reach 0.11–0.26 µM, whereas local bone surface concentrations are modestly higher due to rapid skeletal uptake (Bolland et al., 2012; Qaseem et al., 2017; Yarom et al., 2019). In oncology patients receiving repeated high-dose regimens, local concentrations at bone remodeling sites can transiently reach low micromolar levels (0.4–4.6 µM) (Merigo et al., 2005). Our *in vitro* concentration range (0.01–10 µM) thus encompasses both systemic and early bone exposures relevant to osteoporosis, which promoted PDLSC osteogenesis without cytotoxicity, as well as higher local exposures typical of oncology settings, which induced cytotoxicity and suppressed osteogenic differentiation. These pharmacokinetic considerations provide a clinically grounded rationale for the observed “double-edged sword” effects of ZA, linking physiologically plausible exposures to both pro-osteogenic and cytotoxic outcomes.

Mechanistically, high-throughput transcriptomic profiling coupled with GO and KEGG analyses pinpointed the Wnt/β-catenin and MAPK signaling pathways as the top enriched cascades in ZOL-treated group. The Wnt/β-catenin pathway is a pivotal regulator of bone remodeling, orchestrating osteoprogenitor proliferation, lineage commitment, and matrix mineralization through the stabilization and nuclear translocation of β-catenin (Nie et al., 2020). Canonical activation of this pathway promotes transcription of osteogenic genes such as RUNX2, ALP, and OCN, thereby facilitating the osteogenic differentiation of PDLSCs (Hu et al., 2023; Gu and Bai, 2024). Notably, dysregulation of Wnt signaling has been implicated in osteoporosis and fracture healing impairment, highlighting its clinical relevance (Yang et al., 2025; Tripathi et al., 2022). Although Wnt/β-catenin and MAPK signaling pathways are classical and highly pleiotropic, extensive evidence in bone biology establishes them as central integrators of osteogenic differentiation (Liu et al., 2025; Wang et al., 2023). Canonical Wnt/β-catenin signaling promotes osteoblast lineage commitment and matrix mineralization through β-catenin stabilization, nuclear translocation, and transcriptional activation of osteogenic regulators such as RUNX2 and ALP, whereas disruption of this pathway impairs bone formation and favors alternative mesenchymal fates (Arioka et al., 2013; Alexeeva et al., 2023). In parallel, MAPK cascades—including p38 and JNK—transduce extracellular and intracellular cues to osteogenic programs and functionally cooperate with Wnt signaling to enhance osteogenic gene expression and maturation (Fathi and Farahzadi, 2017; Hu et al., 2016).

In this study, we demonstrate that zoledronate regulates these pathways in a dose-dependent manner: low-dose ZOL induces coordinated activation of Wnt/β-catenin and MAPK signaling, thereby favoring osteogenesis, whereas high-dose ZOL suppresses both pathways in parallel with cytotoxic phenotypes. This divergence is consistent with prior evidence showing that excessive inhibition of the mevalonate pathway by nitrogen-containing bisphosphonates disrupts small GTPase prenylation, alters cytoskeletal organization, and attenuates downstream β-catenin and MAPK signaling (Wrobel et al., 2025). Thus, rather than acting as isolated or nonspecific pathways, Wnt/β-catenin and MAPK signaling serve as convergent nodes that translate bisphosphonate exposure into context-dependent osteogenic or cytotoxic outcomes (Mao et al., 2016; Li et al., 2018). While the identification of all upstream modulators and downstream effectors is beyond the scope of the present study, our findings establish a mechanistically coherent framework in which dose-dependent engagement of Wnt/β-catenin–MAPK signaling underlies the “double-edged sword” effect of zoledronate on PDLSC fate. Further dissection of specific molecular intermediates will be pursued in future studies.

From a translational perspective, our findings support the concept that controlled, localized delivery of low-dose ZOL could be strategically employed to enhance periodontal bone regeneration. Such an approach could circumvent the systemic accumulation associated with long-term BP therapy, thereby reducing MRONJ risk. For instance, incorporating ZOL into biodegradable scaffolds, hydrogels, or nanoparticle delivery systems could provide sustained local release at low concentrations, maintaining osteogenic stimulation while minimizing systemic exposure.

However, the therapeutic exploitation of BPs in this context requires careful consideration of several factors. First, the narrow therapeutic window necessitates precise dosing strategies to avoid tipping the balance toward cytotoxicity (Merugu et al., 2023). Second, the inflammatory and microbial environment of the periodontium *in vivo* may modulate BP effects, potentially altering the signaling balance observed under controlled experimental conditions (Sun et al., 2023). Third, patient-specific factors such as prior BP exposure, systemic comorbidities, and genetic variability in drug metabolism could influence both efficacy and safety (James et al., 2016).

Our study also has limitations. The *in vivo* bone formation assays were performed in ectopic sites, which do not fully replicate the anatomical, vascular, and microbial conditions of periodontal defects. Additionally, we did not assess the long-term stability of ZOL-induced bone or its integration with native tissue. The mechanistic analysis, while revealing key pathway activations, was primarily based on protein phosphorylation and could be strengthened by genetic knockdown or pharmacological inhibition studies to establish causal relationships. Furthermore, the potential involvement of other pathways, such as PI3K/Akt or NF-κB, in mediating ZOL’s effects warrants further exploration.

Future research should prioritize orthotopic periodontal defect models, ideally under conditions that mimic clinical scenarios such as periodontitis or post-extraction healing. This would allow a more accurate assessment of the interplay between ZOL-induced osteogenesis and the inflammatory microenvironment. In parallel, advances in biomaterial engineering—particularly the design of smart scaffolds or nanocarriers enabling controlled, spatiotemporal release of zoledronate—hold substantial translational promise for periodontal bone regeneration. Such strategies could not only maximize the pro-regenerative effects of low-dose ZA while mitigating cytotoxicity, but also pave the way for precision therapeutics that restore functional periodontal tissue in clinical settings.

## Conclusions

**FIGURE 7:**
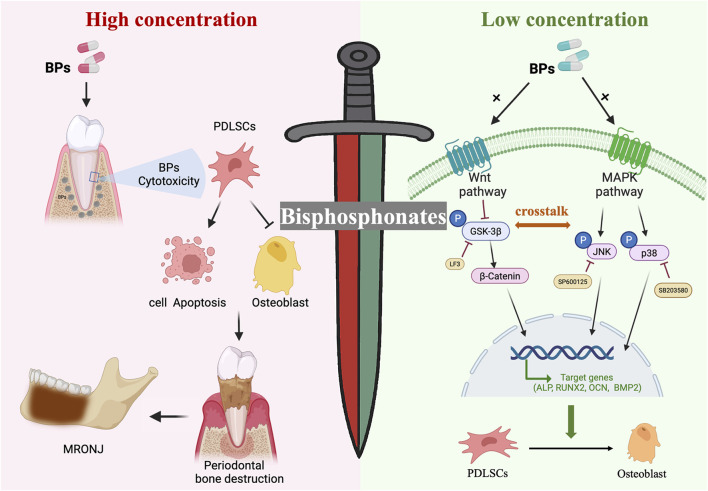
Schematic of the double-edged-sword effect: low concentrations BPs significantly enhanced osteogenic differentiation of periodontal ligament stem cells via crosstalk between the Wnt/β-catenin and MAPK signaling pathways, whereas higher concentrations BPs suppressed proliferation, induced apoptosis, and reduced osteogenesis.

This study revealed that BPs exert a double-edged-sword effect: low-dose BPs promotes PDLSC osteogenesis via coordinated activation of Wnt/β-catenin and MAPK signaling pathways, whereas high-dose induces cytotoxicity. These insights highlight the necessity of precise dosing to harness regenerative benefits while mitigating MRONJ risk, thereby providing important mechanistic and preclinical evidence to guide the rational and safe clinical application of BPs in oral and maxillofacial medicine (Figure 7).

## Data Availability

The authors acknowledge that the data presented in this study must be deposited and made publicly available in an acceptable repository, prior to publication. Frontiers cannot accept a manuscript that does not adhere to our open data policies.

## References

[B49] Açil Y. Moller B. Niehoff P. Rachko K. Gassling V. Wiltfang J. (2012). The cytotoxic effects of three different bisphosphonates in-vitro on human gingival fibroblasts, osteoblasts and osteogenic sarcoma cells. J. Craniomaxillofac. Surg. 40 (8), e229–e235. 10.1016/j.jcms.2011.10.024 22082730

[B1] Akoury E. Ramirez Garcia Luna A. S. Ahangar P. Gao X. Zolotarov P. Weber M. H. (2019). Anti-tumor effects of low dose zoledronate on lung cancer-induced spine metastasis. J. Clin. Med. 8. 10.3390/jcm8081212 31416169 PMC6722631

[B2] Alexeeva E. I. Dvoryakovskaya T. M. Tsulukiya I. T. Kondrateva N. M. Solomatina N. M. Kondratiev G. V. (2023). Juvenile idiopathic arthritis with systemic onset with inflammatory bone lesions: two case reports of patients successfully treated with canakinumab and experience gained from literature. Front. Pediatr. 11, 1163483. 10.3389/fped.2023.1163483 37325364 PMC10266530

[B3] Alsalleeh F. Keippel J. Adams L. Bavitz B. (2014). Bisphosphonate-associated osteonecrosis of jaw reoccurrence after methotrexate therapy: a case report. J. Endod. 40, 1505–1507. 10.1016/j.joen.2014.01.035 25146044

[B4] Arioka M. Takahashi-Yanaga F. Sasaki M. Yoshihara T. Morimoto S. Takashima A. (2013). Acceleration of bone development and regeneration through the Wnt/beta-catenin signaling pathway in mice heterozygously deficient for GSK-3beta. Biochem. Biophys. Res. Commun. 440, 677–682. 10.1016/j.bbrc.2013.09.126 24099767

[B5] Bertoldo F. Eller-Vainicher C. Fusco V. Mauceri R. Pepe J. Bedogni A. (2025). Medication related osteonecrosis (MRONJ) in the management of CTIBL in breast and prostate cancer patients. J. Bone Oncol. 50, 100656. 10.1016/j.jbo.2024.100656 39807373 PMC11728904

[B6] Bolland M. J. Grey A. Horne A. M. Briggs S. E. Thomas M. G. Ellis-Pegler R. B. (2012). Effects of intravenous zoledronate on bone turnover and bone density persist for at least five years in HIV-Infected men. J. Clin. Endocrinol. Metab. 97, 1922–1928. 10.1210/jc.2012-1424 22419728

[B50] Budzinska A. Galganski L. Jarmuszkiewicz W. (2023). The bisphosphonates alendronate and zoledronate induce adaptations of aerobic metabolism in permanent human endothelial cells. Sci. Rep. 13 (1), 16205. 10.1038/s41598-023-43377-3 37758809 PMC10533870

[B7] Cai Y. Gao T. Fu S. Sun P. (2018). Development of zoledronic acid functionalized hydroxyapatite loaded polymeric nanoparticles for the treatment of osteoporosis. Exp. Ther. Med. 16, 704–710. 10.3892/etm.2018.6263 30116324 PMC6090242

[B8] Chen T. Berenson J. Vescio R. Swift R. Gilchick A. Goodin S. (2002). Pharmacokinetics and pharmacodynamics of zoledronic acid in cancer patients with bone metastases. J. Clin. Pharmacol. 42, 1228–1236. 10.1177/009127002762491316 12412821

[B9] Cremers S. Ebetino F. H. Phipps R. (2020). On the pharmacological evaluation of bisphosphonates in humans. Bone 139, 115501. 10.1016/j.bone.2020.115501 32599224 PMC7483926

[B53] Dunford J. E. Rogers M. J. Ebetino F. H. Phipps R. J. Coxon F. P. (2006). Coxon: inhibition of protein prenylation by bisphosphonates causes sustained activation of Rac, Cdc42, and Rho GTPases. J. Bone. Miner. Res. 21 (5), 684–694. 10.1359/jbmr.060118 16734383

[B10] Endo Y. Kumamoto H. Nakamura M. Sugawara S. Takano-Yamamoto T. Sasaki K. (2017). Underlying mechanisms and therapeutic strategies for bisphosphonate-related Osteonecrosis of the Jaw (BRONJ). Biol. Pharm. Bull. 40, 739–750. 10.1248/bpb.b16-01020 28566618

[B11] Fathi E. Farahzadi R. (2017). Enhancement of osteogenic differentiation of rat adipose tissue-derived mesenchymal stem cells by zinc sulphate under electromagnetic field via the PKA, ERK1/2 and Wnt/beta-catenin signaling pathways. PLoS One 12, e0173877. 10.1371/journal.pone.0173877 28339498 PMC5365128

[B52] Gadelha A. P. R. Brigagao C. M. da Silva M. B. Rodrigues A. B. M. Guimaraes A. C. R. Paiva F. (2020). Henriques: insights about the structure of farnesyl diphosphate synthase (FPPS) and the activity of bisphosphonates on the proliferation and ultrastructure of Leishmania and Giardia. Parasit Vectors 13 (1), 168. 10.1186/s13071-020-04019-z 32248823 PMC7132869

[B12] Gu Y. Bai Y. (2024). Osteogenic effect of crocin in human periodontal ligament stem cells via Wnt/beta-catenin signaling. Oral Dis. 30, 1429–1438. 10.1111/odi.14523 36705490

[B51] Guirguis R. H. Tan L. P. Hicks R. M. Hasan A. Duong T. D. Hu X. (2023). Celentano: in vitro cytotoxicity of antiresorptive and antiangiogenic compounds on oral tissues contributing to MRONJ: systematic review. Biomol. 13 (6). 10.3390/biom13060973 37371553 PMC10296421

[B13] Hu H. Chen M. Dai G. Du G. Wang X. He J. (2016). An inhibitory role of osthole in rat MSCs osteogenic differentiation and proliferation via Wnt/beta-Catenin and Erk1/2-MAPK pathways. Cell Physiol. Biochem. 38, 2375–2388. 10.1159/000445590 27300751

[B14] Hu Y. Wang Z. Fan C. Gao P. Wang W. Xie Y. (2023). Human gingival mesenchymal stem cell-derived exosomes cross-regulate the Wnt/beta-catenin and NF-kappaB signalling pathways in the periodontal inflammation microenvironment. J. Clin. Periodontol. 50, 796–806. 10.1111/jcpe.13798 36843393

[B15] James N. D. Pirrie S. J. Pope A. M. Barton D. Andronis L. Goranitis I. (2016). Clinical outcomes and survival following treatment of metastatic castrate-refractory prostate cancer with docetaxel alone or with Strontium-89, zoledronic acid, or both: the TRAPEZE randomized clinical trial. JAMA Oncol. 2, 493–499. 10.1001/jamaoncol.2015.5570 26794729

[B16] Jiang A. Zhang Z. Qiu X. Guo Q. (2024). Medication-related osteonecrosis of the jaw (MRONJ): a review of pathogenesis hypothesis and therapy strategies. Arch. Toxicol. 98, 689–708. 10.1007/s00204-023-03653-7 38155341

[B17] Kishimoto H. Noguchi K. Takaoka K. (2019). Novel insight into the management of bisphosphonate-related osteonecrosis of the jaw (BRONJ). Jpn. Dent. Sci. Rev. 55, 95–102. 10.1016/j.jdsr.2018.09.002 31193410 PMC6526304

[B18] Lee K. Kim K. Kim J. Y. Kim J. W. Kang Y. H. Kim Y. H. (2025). Mechanisms underlying medication-related osteonecrosis of the jaw. Oral Dis. 31, 1073–1083. 10.1111/odi.15198 39552606 PMC12022389

[B19] Li X. Zheng Y. Zheng Y. Huang Y. Zhang Y. Jia L. (2018). Circular RNA CDR1as regulates osteoblastic differentiation of periodontal ligament stem cells via the miR-7/GDF5/SMAD and p38 MAPK signaling pathway. Stem Cell Res. Ther. 9, 232. 10.1186/s13287-018-0976-0 30170617 PMC6119336

[B20] Limones A. Saez-Alcaide L. M. Diaz-Parreno S. A. Helm A. Bornstein M. M. Molinero-Mourelle P. (2020). Medication-related osteonecrosis of the jaws (MRONJ) in cancer patients treated with denosumab vs. zoledronic acid: a systematic review and meta-analysis. Med. Oral Patol. Oral Cir. Bucal 25, e326–e336. 10.4317/medoral.23324 32271321 PMC7211372

[B21] Liu X. Zhao W. Peng Y. Liu N. Liu Q. (2025). The relationship between MAPK signaling pathways and osteogenic differentiation of periodontal ligament stem cells: a literature review. PeerJ 13, e19193. 10.7717/peerj.19193 40183050 PMC11967421

[B22] Mao C. Y. Wang Y. G. Zhang X. Zheng X. Y. Tang T. T. Lu E. Y. (2016). Double-edged-sword effect of IL-1beta on the osteogenesis of periodontal ligament stem cells via crosstalk between the NF-kappaB, MAPK and BMP/Smad signaling pathways. Cell Death Dis. 7, e2296. 10.1038/cddis.2016.204 27415426 PMC4973347

[B23] Merigo E. Manfredi M. Meleti M. Corradi D. Vescovi P. (2005). Jaw bone necrosis without previous dental extractions associated with the use of bisphosphonates (pamidronate and zoledronate): a four-case report. J. Oral Pathol. Med. 34, 613–617. 10.1111/j.1600-0714.2005.00351.x 16202082

[B24] Merugu C. Sahoo J. Kamalanathan S. Ramkumar G. Reddy S. V. B. Kar S. S. (2023). Effect of a single dose of zoledronic acid on bone mineral density and trabecular bone score in Indian postmenopausal osteoporotic women with and without type 2 diabetes mellitus - a prospective cohort pilot study. Endocrine 82, 171–180. 10.1007/s12020-023-03432-5 37368233

[B25] Moreno Rabie C. Garcia-Larrain S. Contreras D. E. Medina D. Cabello-Salazar I. Fontenele R. C. (2023). How does the clinical and tomographic appearance of MRONJ influences its treatment prognosis? Dentomaxillofac Radiol. 52, 20230304. 10.1259/dmfr.20230304 37870051 PMC10968764

[B26] Moshaverinia A. Chen C. Xu X. Akiyama K. Ansari S. Zadeh H. H. (2014). Bone regeneration potential of stem cells derived from periodontal ligament or gingival tissue sources encapsulated in RGD-modified alginate scaffold. Tissue Eng. Part A 20, 611–621. 10.1089/ten.TEA.2013.0229 24070211 PMC3926152

[B27] Nie F. Zhang W. Cui Q. Fu Y. Li H. Zhang J. (2020). Kaempferol promotes proliferation and osteogenic differentiation of periodontal ligament stem cells via Wnt/beta-catenin signaling pathway. Life Sci. 258, 118143. 10.1016/j.lfs.2020.118143 32717269

[B28] Qaseem A. Forciea M. A. Mclean R. M. Denberg T. D. Barry M. J. Cooke M. (2017). Treatment of low bone density or osteoporosis to prevent fractures in men and women: a clinical practice guideline update from the American college of physicians. Ann. Intern. Med. 166, 818–839. 10.7326/M15-1361 28492856

[B29] Raccor B. S. Sun J. Lawrence R. F. Li L. Zhang H. Somerman M. J. (2013). Quantitation of zoledronic acid in murine bone by liquid chromatography coupled with tandem mass spectrometry. J. Chromatogr. B Anal. Technol. Biomed. Life Sci. 935, 54–60. 10.1016/j.jchromb.2013.07.019 23954589 PMC3869204

[B30] Roato I. Mauceri R. Notaro V. Genova T. Fusco V. Mussano F. (2023). Immune dysfunction in medication-related osteonecrosis of the jaw. Int. J. Mol. Sci. 24, 7948. 10.3390/ijms24097948 37175652 PMC10177780

[B31] Ruggiero S. L. Dodson T. B. Fantasia J. Goodday R. Aghaloo T. Mehrotra B. (2014). American association of oral and maxillofacial surgeons position paper on medication-related osteonecrosis of the jaw--2014 update. J. Oral Maxillofac. Surg. 72, 1938–1956. 10.1016/j.joms.2014.04.031 25234529

[B32] Ruksakiet K. Jarusriwanna A. Sadaeng W. Laoruengthana A. Sang-Ngoen T. Dhippayom T. (2025). Effects of discontinuing different antiresorptive regimens on medication-related osteonecrosis of the jaw in patients undergoing dental procedures: a systematic review and network meta-analysis. EFORT Open Rev. 10, 258–266. 10.1530/EOR-2024-0133 40326547 PMC12061011

[B33] Skerjanec A. Berenson J. Hsu C. Major P. Miller W. H. Ravera C. (2003). The pharmacokinetics and pharmacodynamics of zoledronic acid in cancer patients with varying degrees of renal function. J. Clin. Pharmacol. 43, 154–162. 10.1177/0091270002239824 12616668

[B34] Srivichit B. Thonusin C. Chattipakorn N. Chattipakorn S. C. (2022). Impacts of bisphosphonates on the bone and its surrounding tissues: mechanistic insights into medication-related osteonecrosis of the jaw. Arch. Toxicol. 96, 1227–1255. 10.1007/s00204-021-03220-y 35199244

[B35] Sun H. Li P. Kong Q. Deng F. Yu X. (2023). Zoledronic acid affects the process of Porphyromonas gingivalis infecting oral mucosal epithelial barrier: an *in-vivo* and *in-vitro* study. Front. Cell Infect. Microbiol. 13, 1104826. 10.3389/fcimb.2023.1104826 37056703 PMC10086244

[B36] Teixeira S. Branco L. Fernandes M. H. Costa-Rodrigues J. (2019). Bisphosphonates and cancer: a relationship beyond the antiresorptive effects. Mini Rev. Med. Chem. 19, 988–998. 10.2174/1389557519666190424163044 31020940

[B37] Tomokiyo A. Wada N. Maeda H. (2019). Periodontal ligament stem cells: regenerative potency in periodontium. Stem Cells Dev. 28, 974–985. 10.1089/scd.2019.0031 31215350

[B38] Tripathi A. K. Rai D. Kothari P. Kushwaha P. Sashidhara K. V. Trivedi R. (2022). Benzofuran pyran hybrid prevents glucocorticoid induced osteoporosis in mice via modulation of canonical Wnt/beta-catenin signaling. Apoptosis 27, 90–111. 10.1007/s10495-021-01702-z 35107658 PMC8808472

[B39] Vassaki M. Lazarou S. Turhanen P. Choquesillo-Lazarte D. Demadis K. D. (2022). Drug-inclusive inorganic-organic hybrid systems for the controlled release of the osteoporosis drug zoledronate. Molecules 27, 6212. 10.3390/molecules27196212 36234745 PMC9572319

[B40] Vuorimies I. Mayranpaa M. K. Valta H. Kroger H. Toiviainen-Salo S. Makitie O. (2017). Bisphosphonate treatment and the characteristics of femoral fractures in children with osteogenesis imperfecta. J. Clin. Endocrinol. Metab. 102, 1333–1339. 10.1210/jc.2016-3745 28323993

[B41] Walter C. Pabst A. Ziebart T. Klein M. Al-Nawas B. (2011). Bisphosphonates affect migration ability and cell viability of HUVEC, fibroblasts and osteoblasts *in vitro* . Oral Dis. 17, 194–199. 10.1111/j.1601-0825.2010.01720.x 20796232

[B42] Wang M. Liu M. Zheng J. Xiong L. Wang P. (2023). Exendin-4 regulates the MAPK and WNT signaling pathways to alleviate the osteogenic inhibition of periodontal ligament stem cells in a high glucose environment. Open Med. (Wars) 18, 20230692. 10.1515/med-2023-0692 37034502 PMC10080709

[B43] Wong C. H. Tsoi K. H. Pu J. J. Jiang N. S. Chan S. S. Y. Loong C. H. N. (2025). Osteoporosis management after the occurrence of medication-related osteonecrosis of the jaw: a 13-Year experience at a tertiary center. Endocrinol. Metab. (Seoul). 40 (6), 974–990. 10.3803/EnM.2024.2262 40509705 PMC12765866

[B44] Wrobel E. Wojdasiewicz P. Mikulska A. Szukiewicz D. (2025). beta-Catenin: a key molecule in osteoblast differentiation. Biomolecules 15 (7), 1043. 10.3390/biom15071043 40723914 PMC12293748

[B45] Yang L. Wang K. Zeng Z. H. Zhao H. E. Bai L. M. (2025). Morroniside improves diabetic osteoporosis via the AGE/RAGE/Wnt/beta-Catenin signaling pathway. Kaohsiung J. Med. Sci. 41, e70063. 10.1002/kjm2.70063 40569797 PMC12520510

[B46] Yarom N. Shapiro C. L. Peterson D. E. Van Poznak C. H. Bohlke K. Ruggiero S. L. (2019). Medication-related osteonecrosis of the jaw: MASCC/ISOO/ASCO clinical practice guideline. J. Clin. Oncol. 37, 2270–2290. 10.1200/JCO.19.01186 31329513

[B47] Zhou Q. Zhao Z. N. Cheng J. T. Zhang B. Xu J. Huang F. (2011). Ibandronate promotes osteogenic differentiation of periodontal ligament stem cells by regulating the expression of microRNAs. Biochem. Biophys. Res. Commun. 404, 127–132. 10.1016/j.bbrc.2010.11.079 21108928

[B48] Ziebart T. Pabst A. Klein M. O. Kammerer P. Gauss L. Brullmann D. (2011). Bisphosphonates: restrictions for vasculogenesis and angiogenesis: inhibition of cell function of endothelial progenitor cells and mature endothelial cells *in vitro* . Clin. Oral Investig. 15, 105–111. 10.1007/s00784-009-0365-2 20024592

