# Supplementary material for: Double-edged-sword effect of bisphosphonates on the osteogenic differentiation of human periodontal ligament stem cells
Source: Front Pharmacol. 2026 Mar 3;17:1752252. doi: 10.3389/fphar.2026.1752252 (PMC12993278; doi:10.3389/fphar.2026.1752252)
Supplement: Supplementary file 1 [file Supplementaryfile1.docx]

**Supplementary Materials for**

**Double-edged-sword effect of bisphosphonates on the osteogenic differentiation of periodontal ligament stem cells**

**Tbale 1**  Primer sequences of human mRNA in quantitative real-time PCR analysis

| Gene | Forward (5'–3') | Reverse (5'–3') |
| --- | --- | --- |
| β-actin | CGACAGGATGCAGAAGGAGA | CATCTGCTGGAAGGTGGACA |
| OCN | CAGGCGCTACCTGTATCAATGGC | GCCGATGTGGTCAGCCAACTC |
| Runx2 | AACAGCAGCAGCAGCAGCAG | GCACCGAGCACAGGAAGTTGG |
| BMP2 | GACGTTGGTCAACTCTGTTAAC | GTCAAGGTACAGCATCGAGAA |
| ALP | GCCTACACGGTCCTCCTATACGG | CACTGCTGACTGCTGCCGATAC |

**Figure 5F** Full uncropped Gels and Blots image.

**
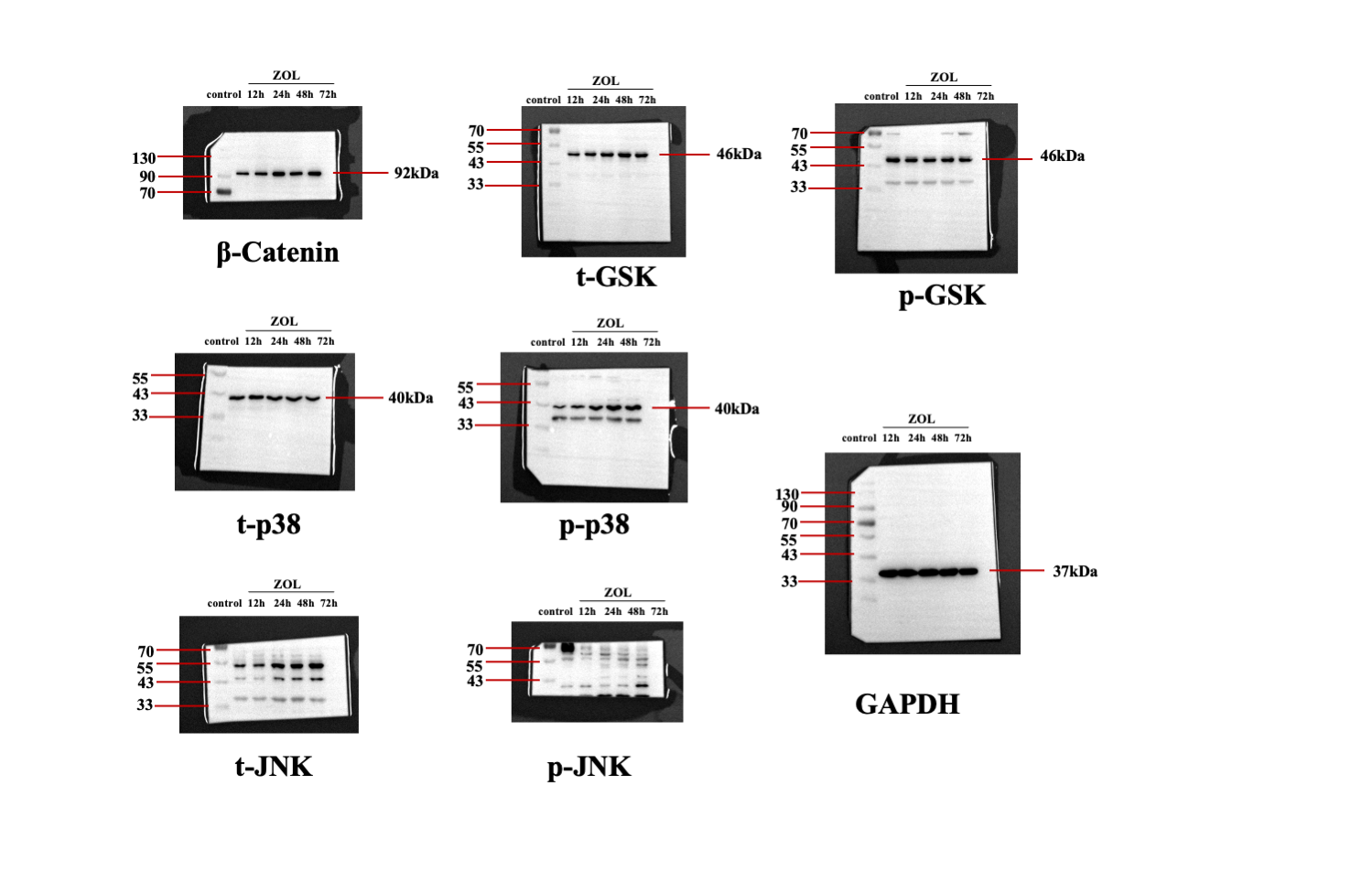
**

**Figure 6A** Full uncropped Gels and Blots image.

**
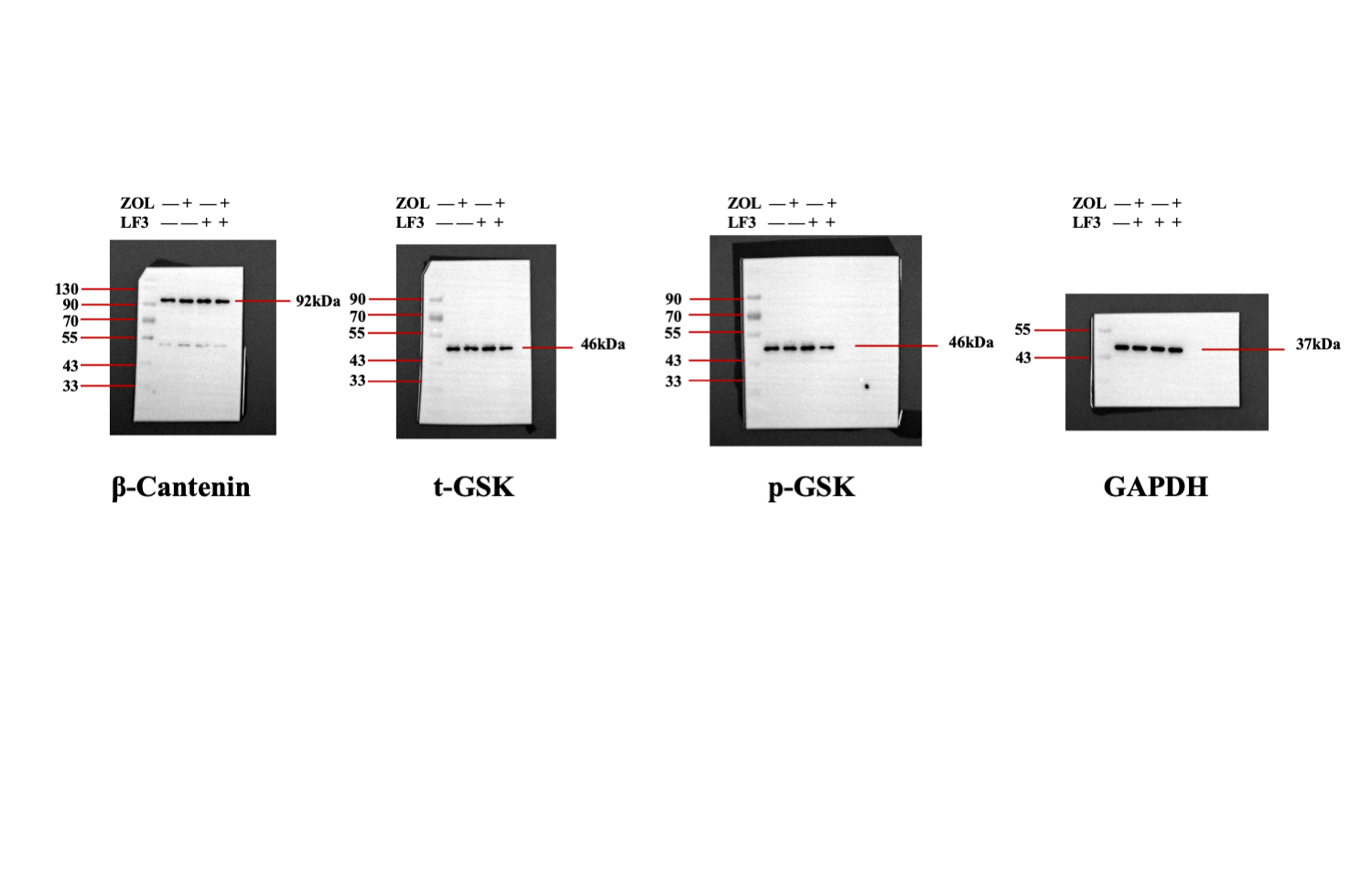
**

**Figure 6B** Full uncropped Gels and Blots image.

**
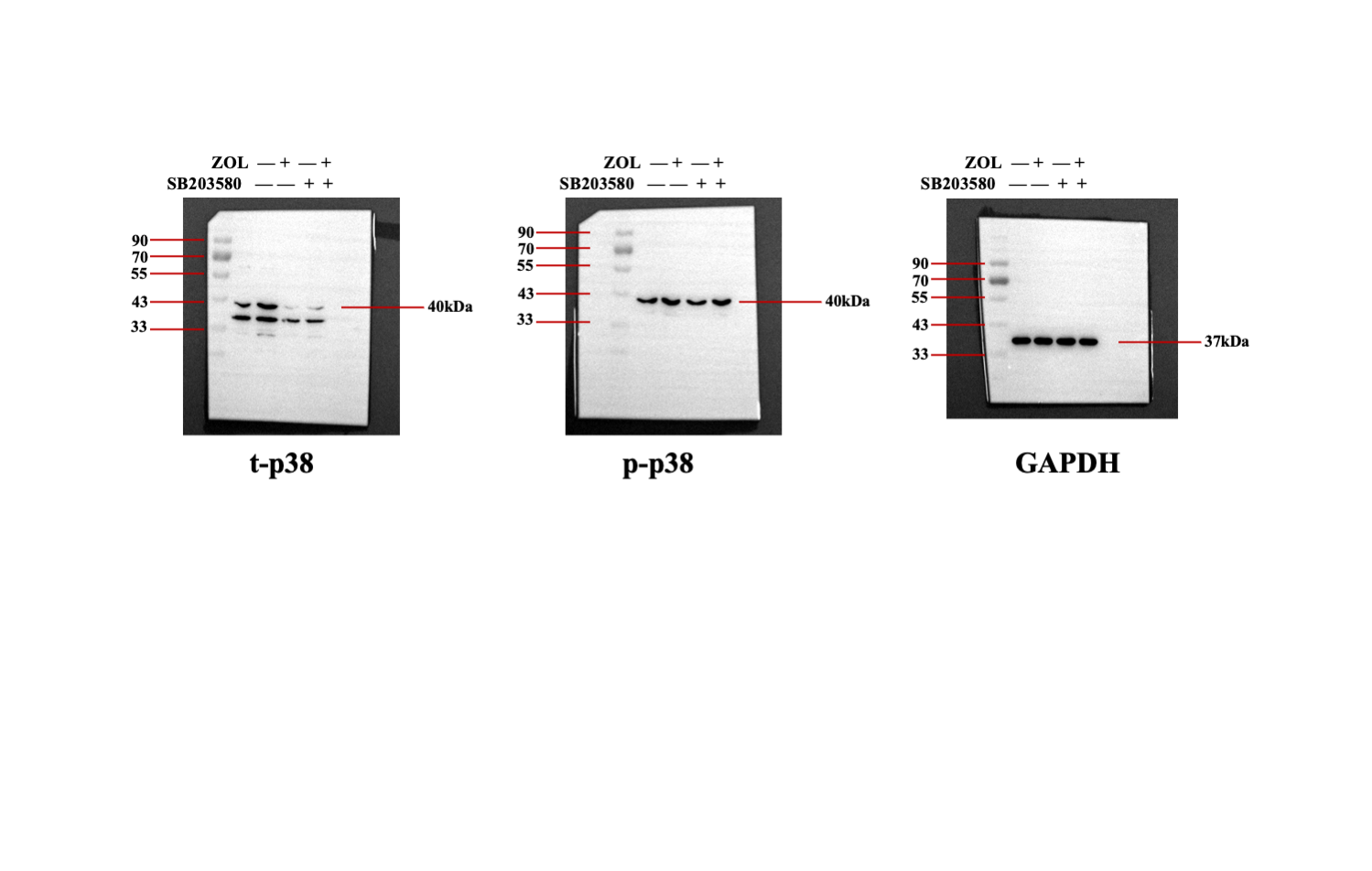
**

**Figure 6C** Full uncropped Gels and Blots image.

**
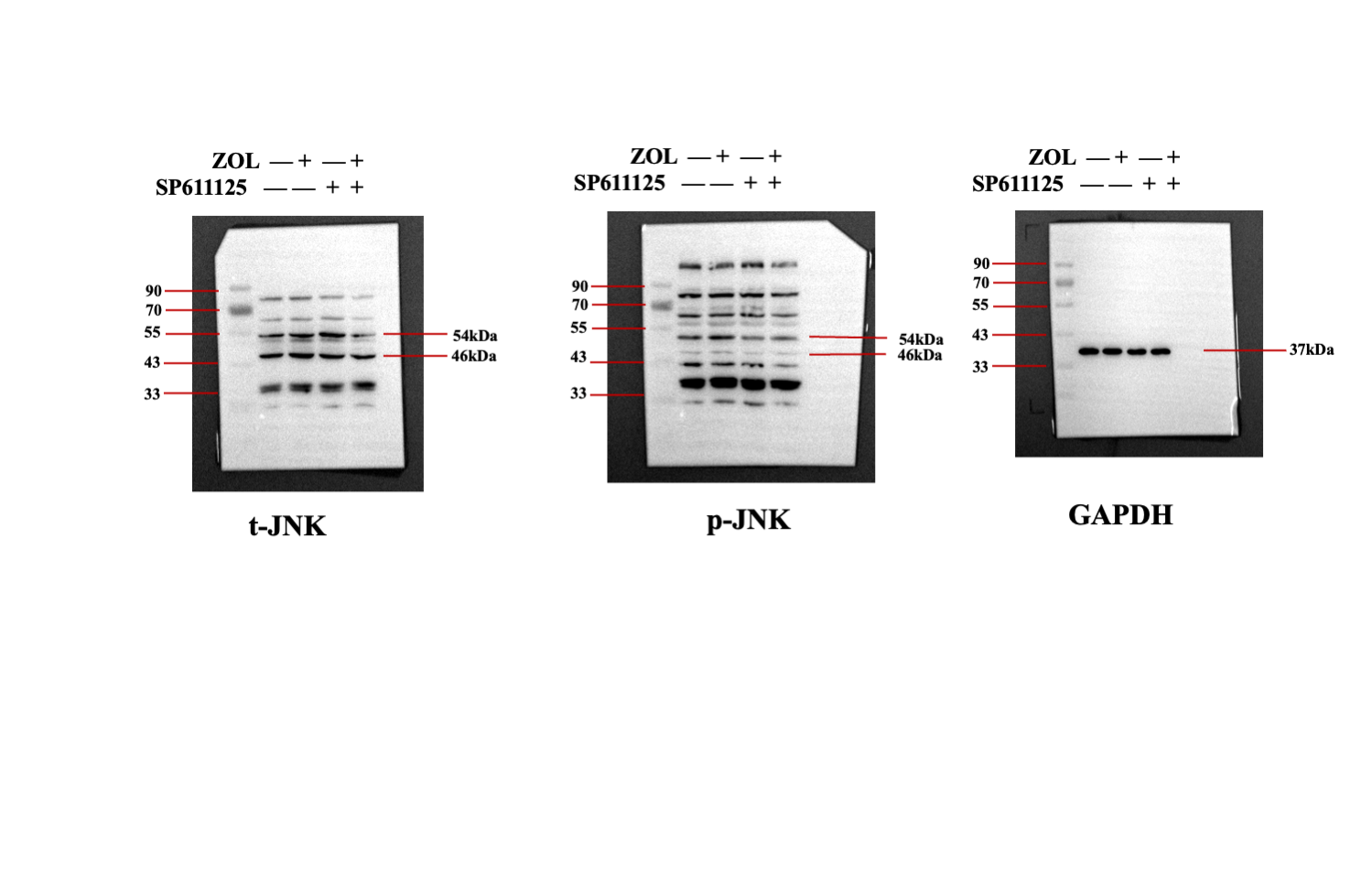
**
